# Supplementary material for: A Humanized Yeast Phenomic Model of Deoxycytidine Kinase to Predict Genetic Buffering of Nucleoside Analog Cytotoxicity
Source: Genes (Basel). 2019 Sep 30;10(10):770. doi: 10.3390/genes10100770 (PMC6826991; doi:10.3390/genes10100770)
Supplement: Supplementary file 1 [file genes-10-00770-s001.zip › Additional_Files/Additional_File7_GOTermHeatmaps.zip!/Additional_File7_GOTermHeatmaps/vesicle_organization.pdf]

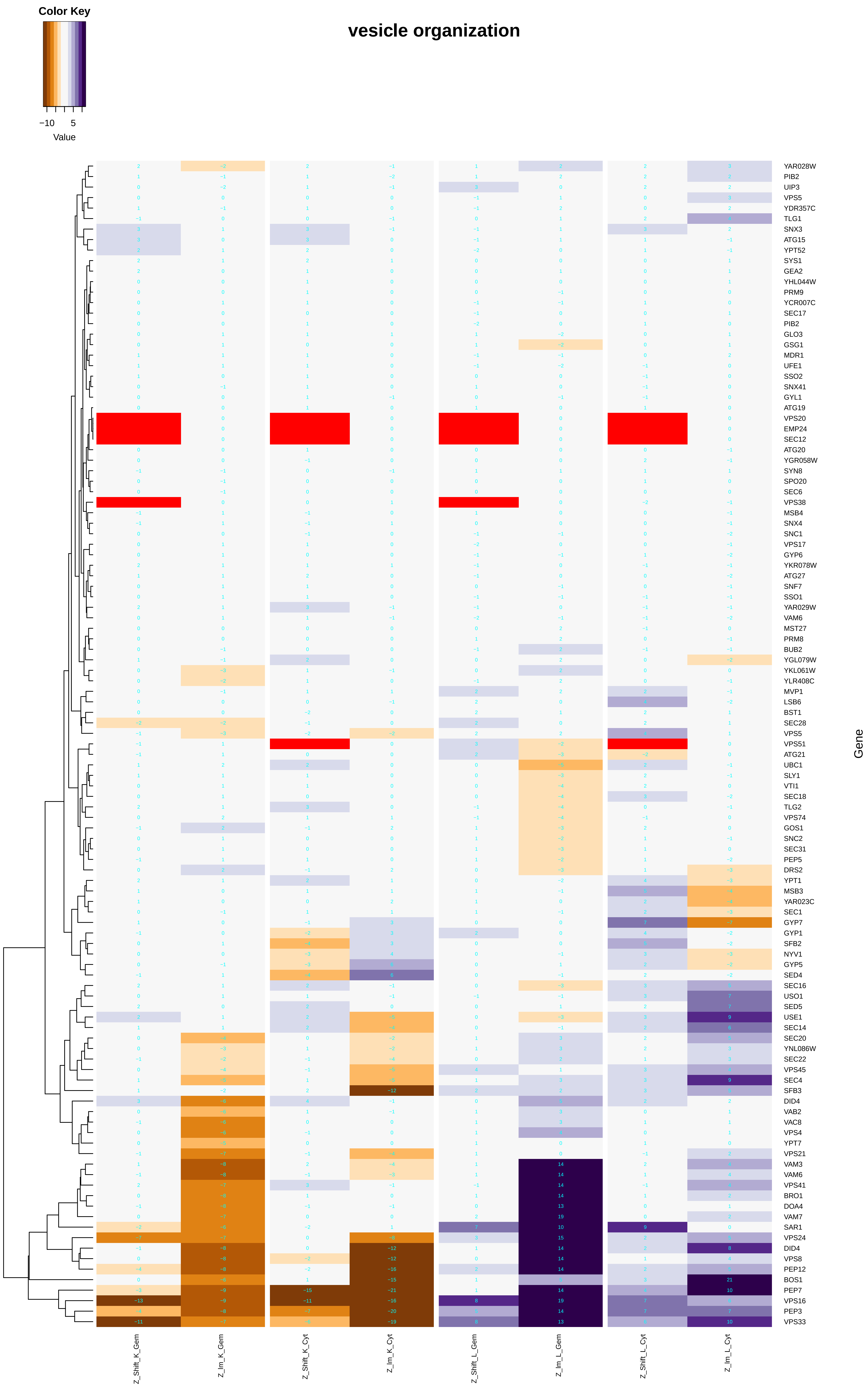

vesicle organization
Color Key
−10
5
Value
Gene
Z_Shift_K_Gem
Z_Im_K_Gem
Z_Shift_K_Cyt
Z_Im_K_Cyt
Z_Shift_L_Gem
Z_Im_L_Gem
Z_Shift_L_Cyt
Z_Im_L_Cyt
YAR028W
PIB2
UIP3
VPS5
YDR357C
TLG1
SNX3
ATG15
YPT52
SYS1
GEA2
YHL044W
PRM9
YCR007C
SEC17
PIB2
GLO3
GSG1
MDR1
UFE1
SSO2
SNX41
GYL1
ATG19
VPS20
EMP24
SEC12
ATG20
YGR058W
SYN8
SPO20
SEC6
VPS38
MSB4
SNX4
SNC1
VPS17
GYP6
YKR078W
ATG27
SNF7
SSO1
YAR029W
VAM6
MST27
PRM8
BUB2
YGL079W
YKL061W
YLR408C
MVP1
LSB6
BST1
SEC28
VPS5
VPS51
ATG21
UBC1
SLY1
VTI1
SEC18
TLG2
VPS74
GOS1
SNC2
SEC31
PEP5
DRS2
YPT1
MSB3
YAR023C
SEC1
GYP7
GYP1
SFB2
NYV1
GYP5
SED4
SEC16
USO1
SED5
USE1
SEC14
SEC20
YNL086W
SEC22
VPS45
SEC4
SFB3
DID4
VAB2
VAC8
VPS4
YPT7
VPS21
VAM3
VAM6
VPS41
BRO1
DOA4
VAM7
SAR1
VPS24
DID4
VPS8
PEP12
BOS1
PEP7
VPS16
PEP3
VPS33

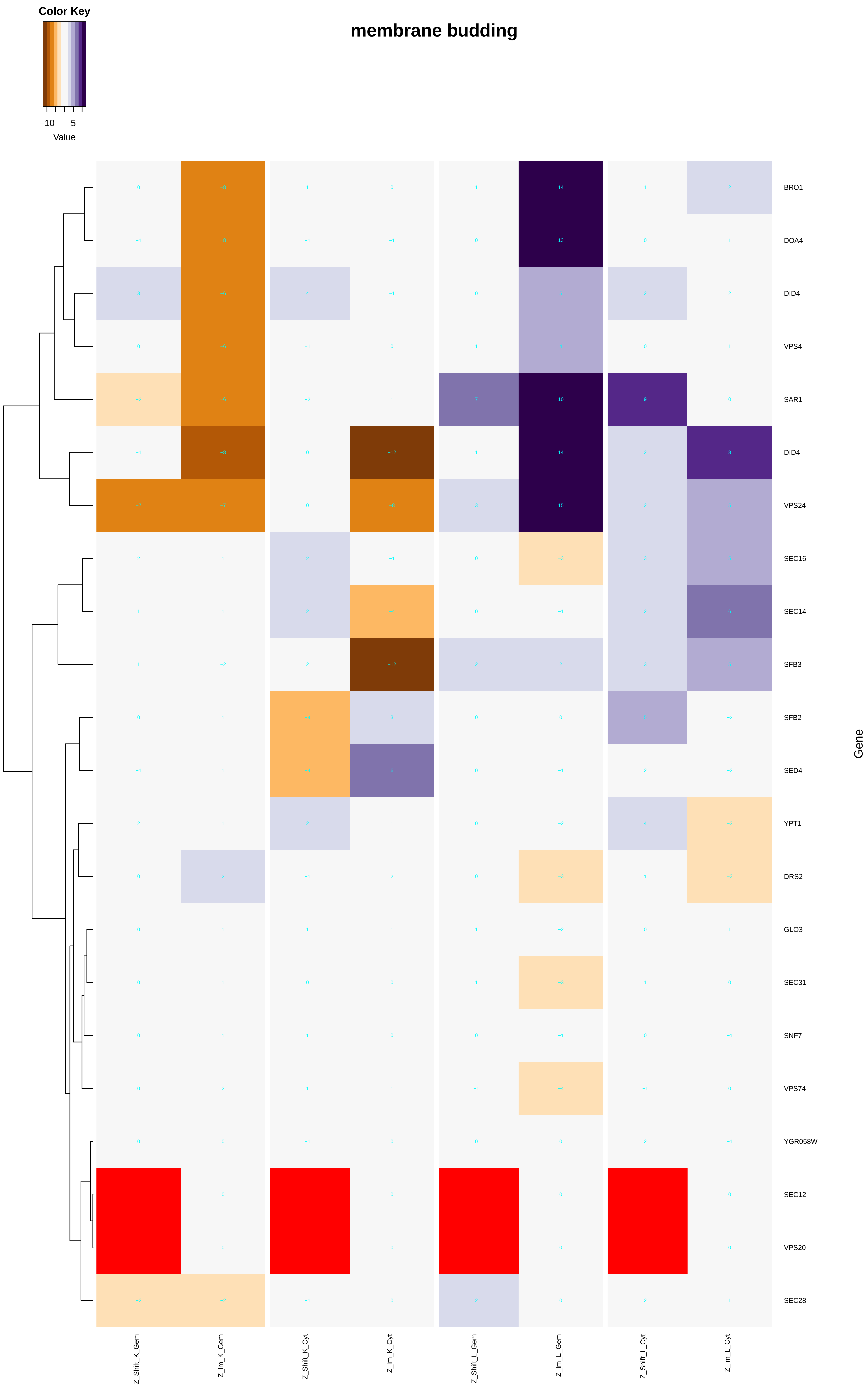
Color Key
−10
5
Value
membrane budding
BRO1
DOA4
DID4
VPS4
SAR1
DID4
VPS24
SEC16
SEC14
SFB3
SFB2
SED4
YPT1
DRS2
GLO3
SEC31
SNF7
VPS74
YGR058W
SEC12
VPS20
SEC28
Gene
Z_Shift_K_Gem
Z_lm_K_Gem
Z_Shift_K_Cyt
Z_lm_K_Cyt
Z_Shift_L_Gem
Z_lm_L_Gem
Z_Shift_L_Cyt
Z_lm_L_Cyt

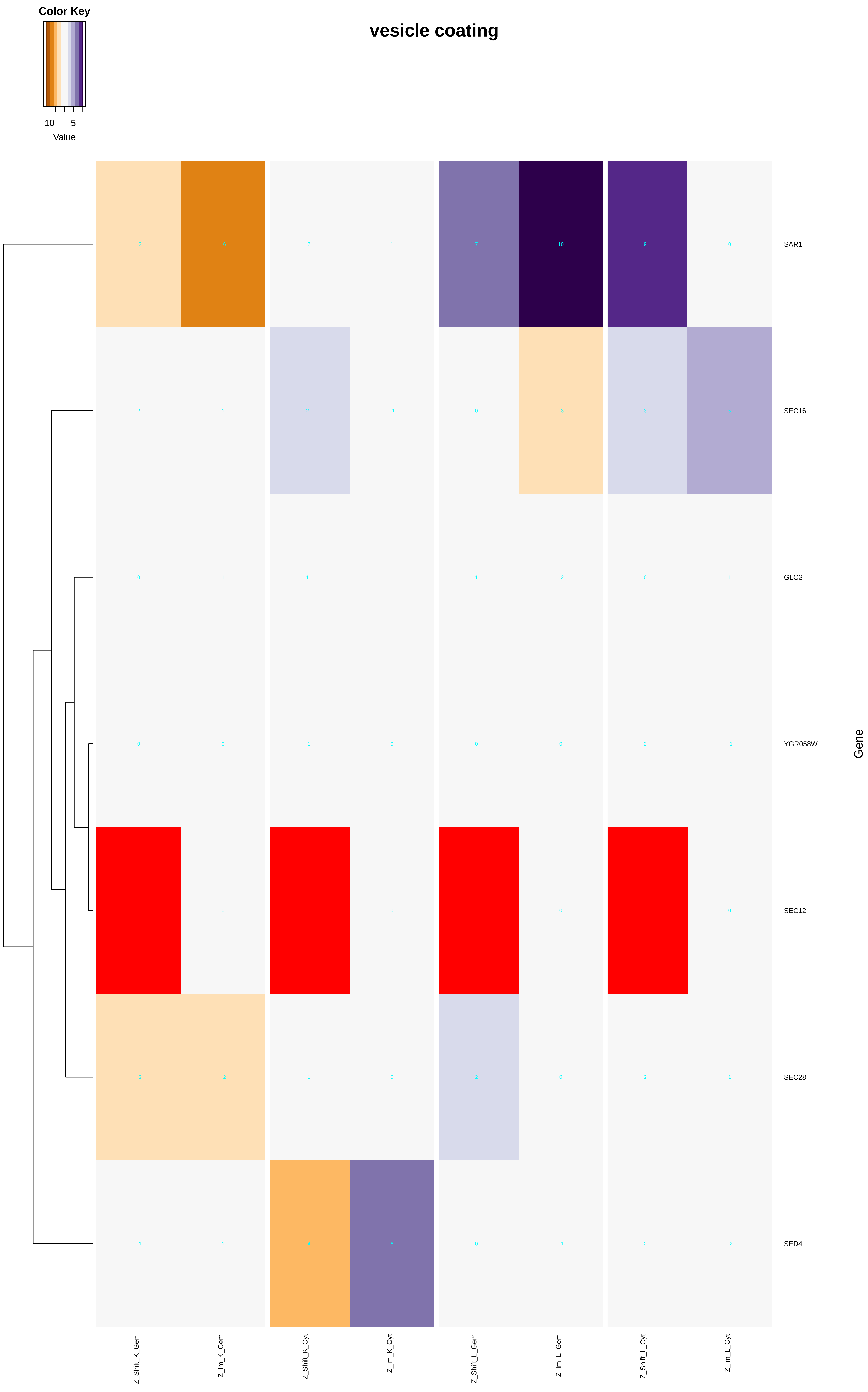

vesicle coating
Color Key
−10
5
Value
SAR1
SEC16
GLO3
YGR058W
SEC12
SEC28
SED4
Gene
Z_Shift_K_Gem
Z_Im_K_Gem
Z_Shift_K_Cyt
Z_Im_K_Cyt
Z_Shift_L_Gem
Z_Im_L_Gem
Z_Shift_L_Cyt
Z_Im_L_Cyt

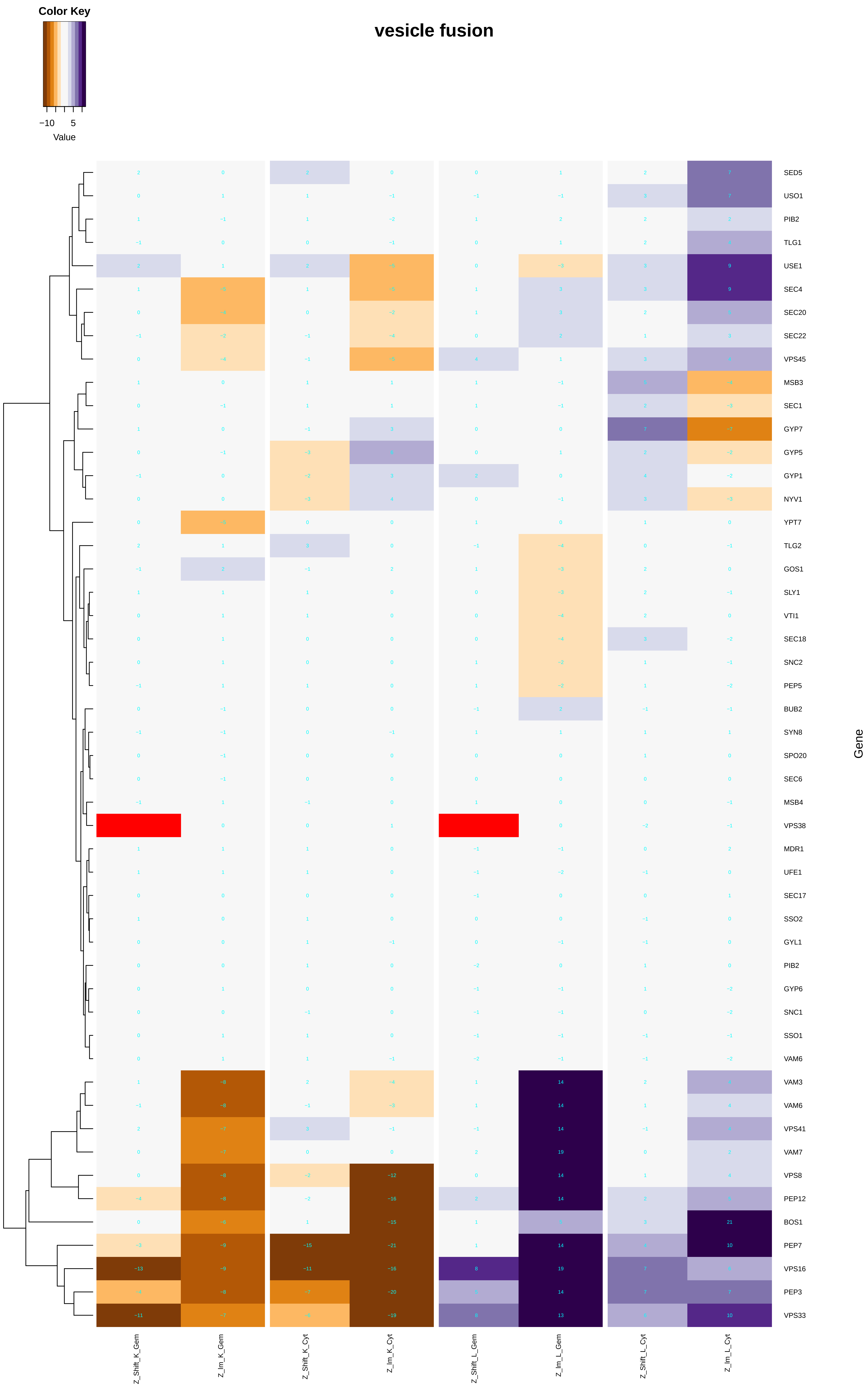
vesicle fusion
Color Key
−10
5
Value
Gene
SED5
USO1
PIB2
TLG1
USE1
SEC4
SEC20
SEC22
VPS45
MSB3
SEC1
GYP7
GYP5
GYP1
NYV1
YPT7
TLG2
GOS1
SLY1
VTI1
SEC18
SNC2
PEP5
BUB2
SYN8
SPO20
SEC6
MSB4
VPS38
MDR1
UFE1
SEC17
SSO2
GYL1
PIB2
GYP6
SNC1
SSO1
VAM6
VAM3
VAM6
VPS41
VAM7
VPS8
PEP12
BOS1
PEP7
VPS16
PEP3
VPS33
Z_Shift_K_Gem
Z_Im_K_Gem
Z_Shift_K_Cyt
Z_Im_K_Cyt
Z_Shift_L_Gem
Z_Im_L_Gem
Z_Shift_L_Cyt
Z_Im_L_Cyt

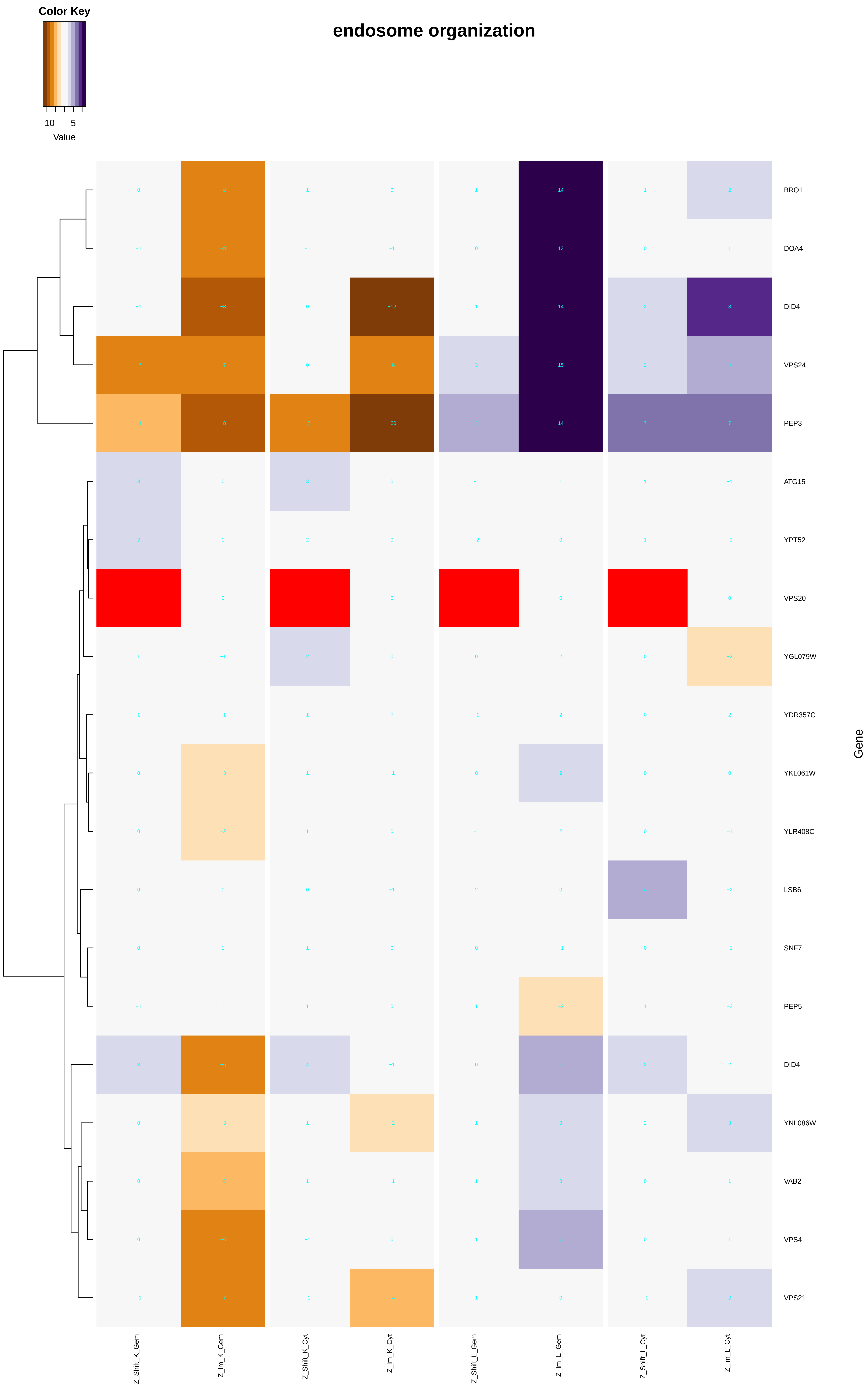
endosome organization
Color Key
−10
5
Value
BRO1
DOA4
DID4
VPS24
PEP3
ATG15
YPT52
VPS20
YGL079W
YDR357C
YKL061W
YLR408C
LSB6
SNF7
PEP5
DID4
YNL086W
VAB2
VPS4
VPS21
Gene
Z_Shift_K_Gem
Z_Im_K_Gem
Z_Shift_K_Cyt
Z_Im_K_Cyt
Z_Shift_L_Gem
Z_Im_L_Gem
Z_Shift_L_Cyt
Z_Im_L_Cyt

Golgi vesicle budding

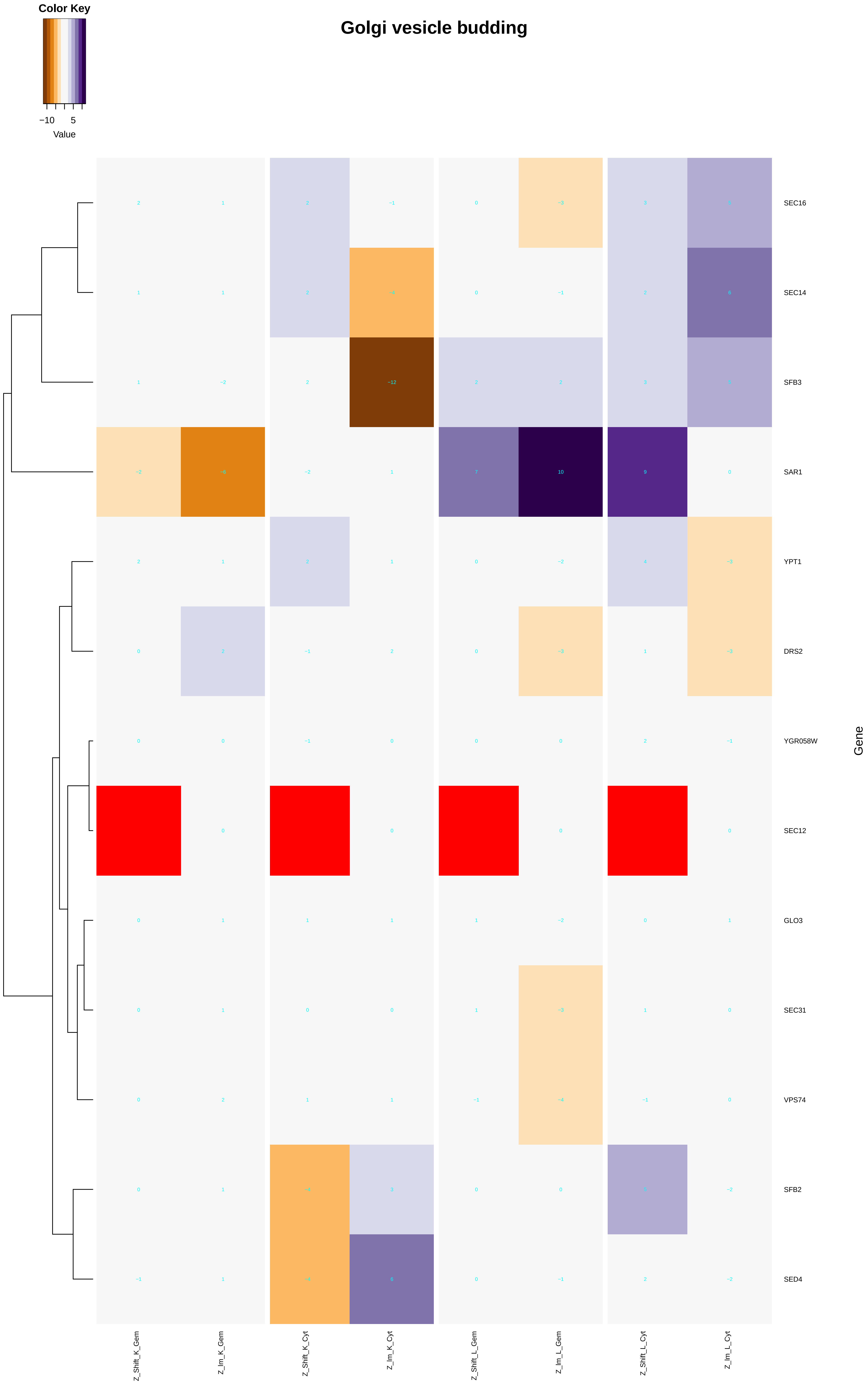

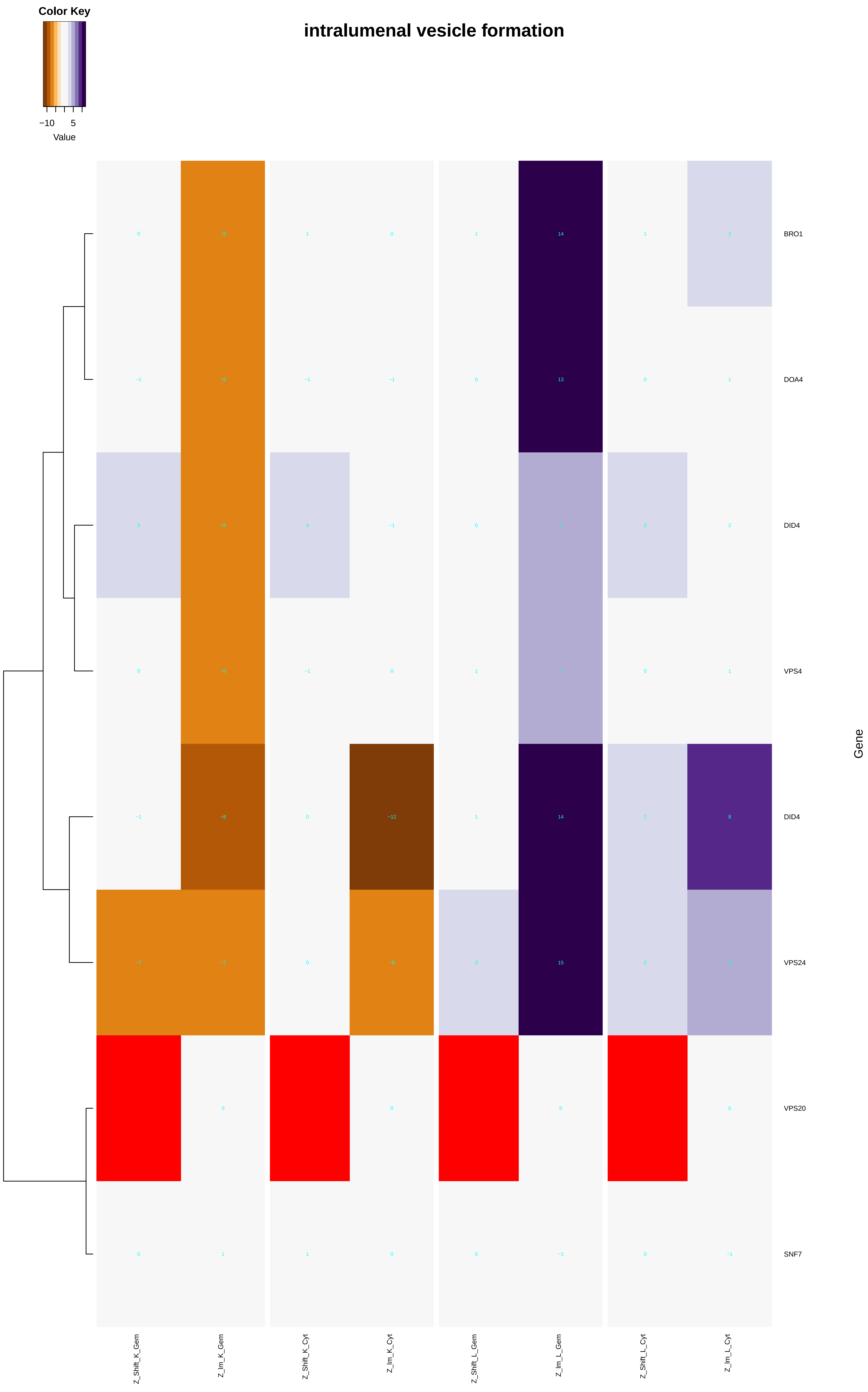
Color Key
−10
5
Value
intralumenal vesicle formation
BRO1
DOA4
DID4
VPS4
DID4
VPS24
VPS20
SNF7
Gene
Z_Shift_K_Gem
Z_Im_K_Gem
Z_Shift_K_Cyt
Z_Im_K_Cyt
Z_Shift_L_Gem
Z_Im_L_Gem
Z_Shift_L_Cyt
Z_Im_L_Cyt

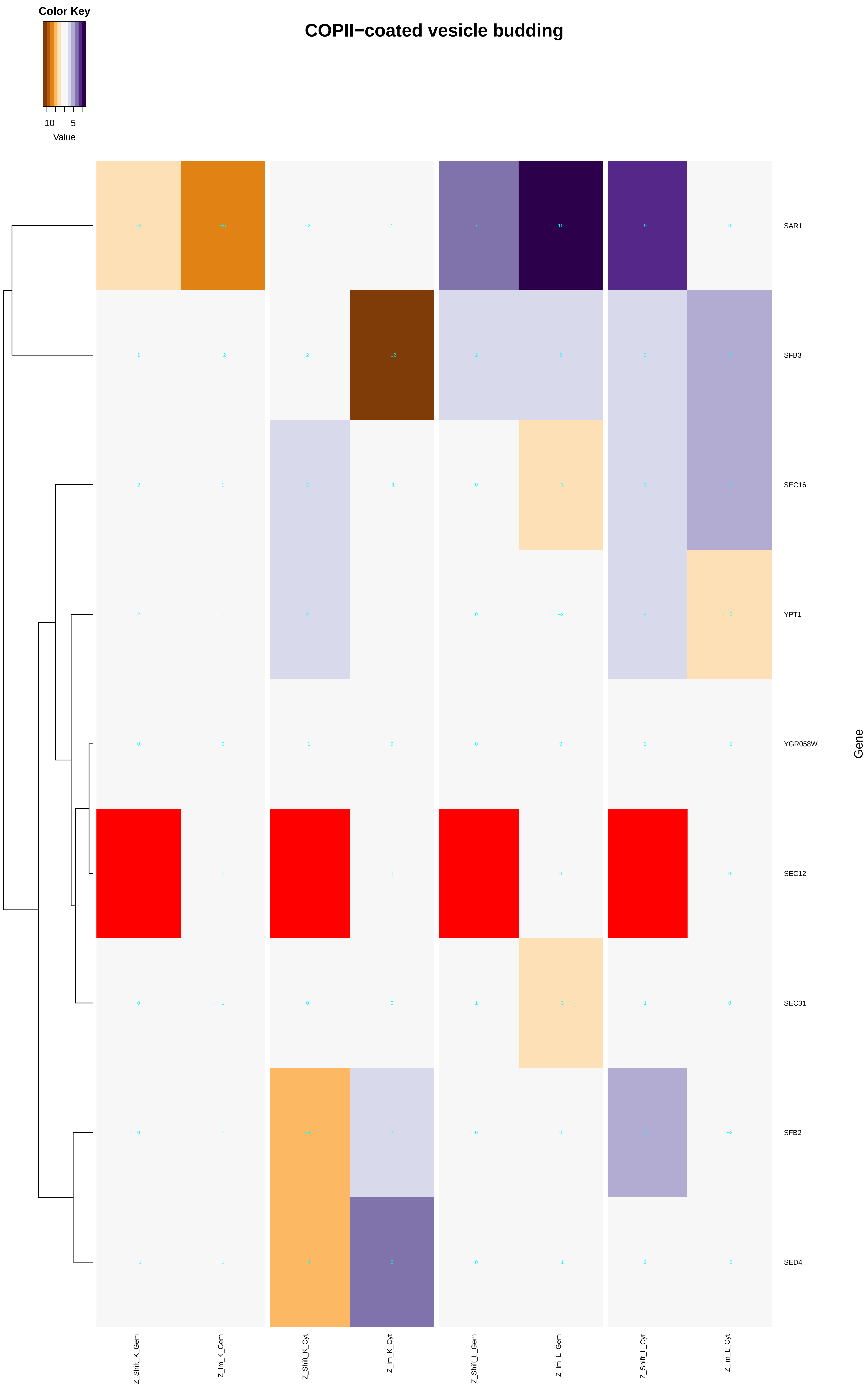

Color Key
−10
5
Value
COPII−coated vesicle budding
SAR1
SFB3
SEC16
YPT1
YGR058W
SEC12
SEC31
SFB2
SED4
Gene
Z_Shift_K_Gem
Z_Im_K_Gem
Z_Shift_K_Cyt
Z_Im_K_Cyt
Z_Shift_L_Gem
Z_Im_L_Gem
Z_Shift_L_Cyt
Z_Im_L_Cyt

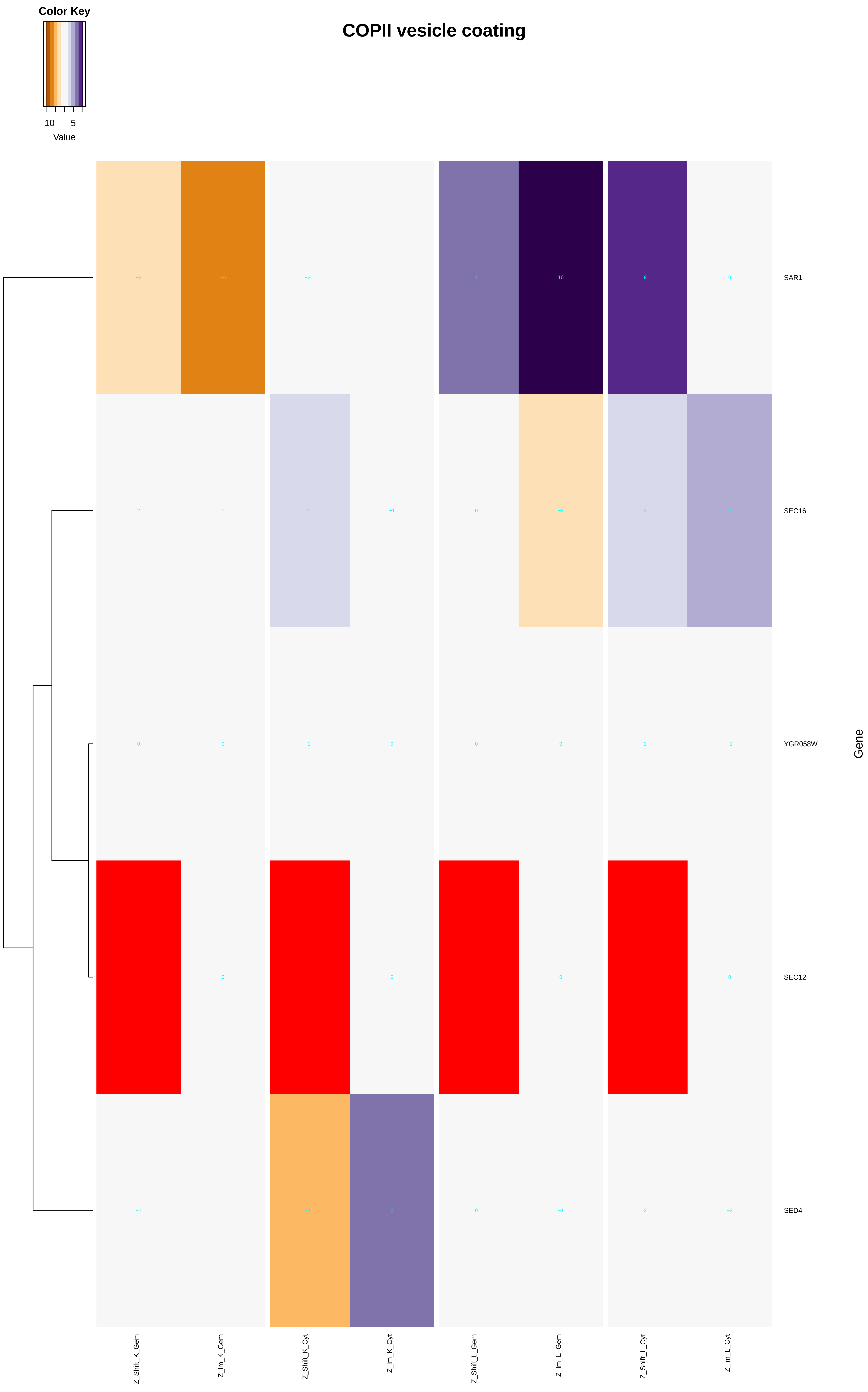
Color Key
−10
5
Value
COPII vesicle coating
SAR1
SEC16
YGR058W
SEC12
SED4
Gene
Z_Shift_K_Gem
Z_Im_K_Gem
Z_Shift_K_Cyt
Z_Im_K_Cyt
Z_Shift_L_Gem
Z_Im_L_Gem
Z_Shift_L_Cyt
Z_Im_L_Cyt

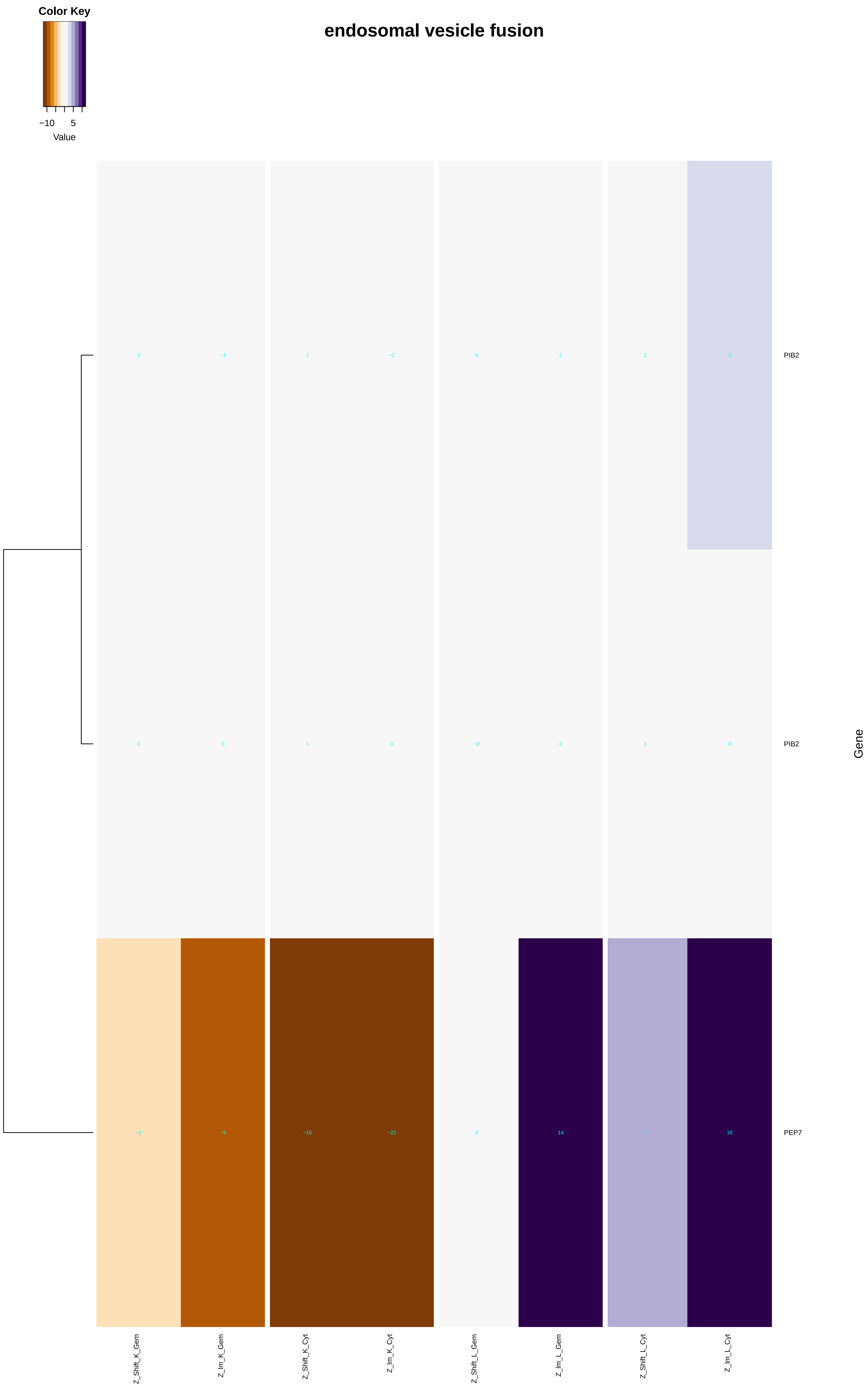
Color Key
−10
5
Value
endosomal vesicle fusion
1
−1
1
−2
1
2
2
2
PIB2
0
0
1
0
−2
0
1
0
PIB2
−3
−9
−15
−21
1
14
10
PEP7
Gene
Z_Shift_K_Gem
Z_Im_K_Gem
Z_Shift_K_Cyt
Z_Im_K_Cyt
Z_Shift_L_Gem
Z_Im_L_Gem
Z_Shift_L_Cyt
Z_Im_L_Cyt

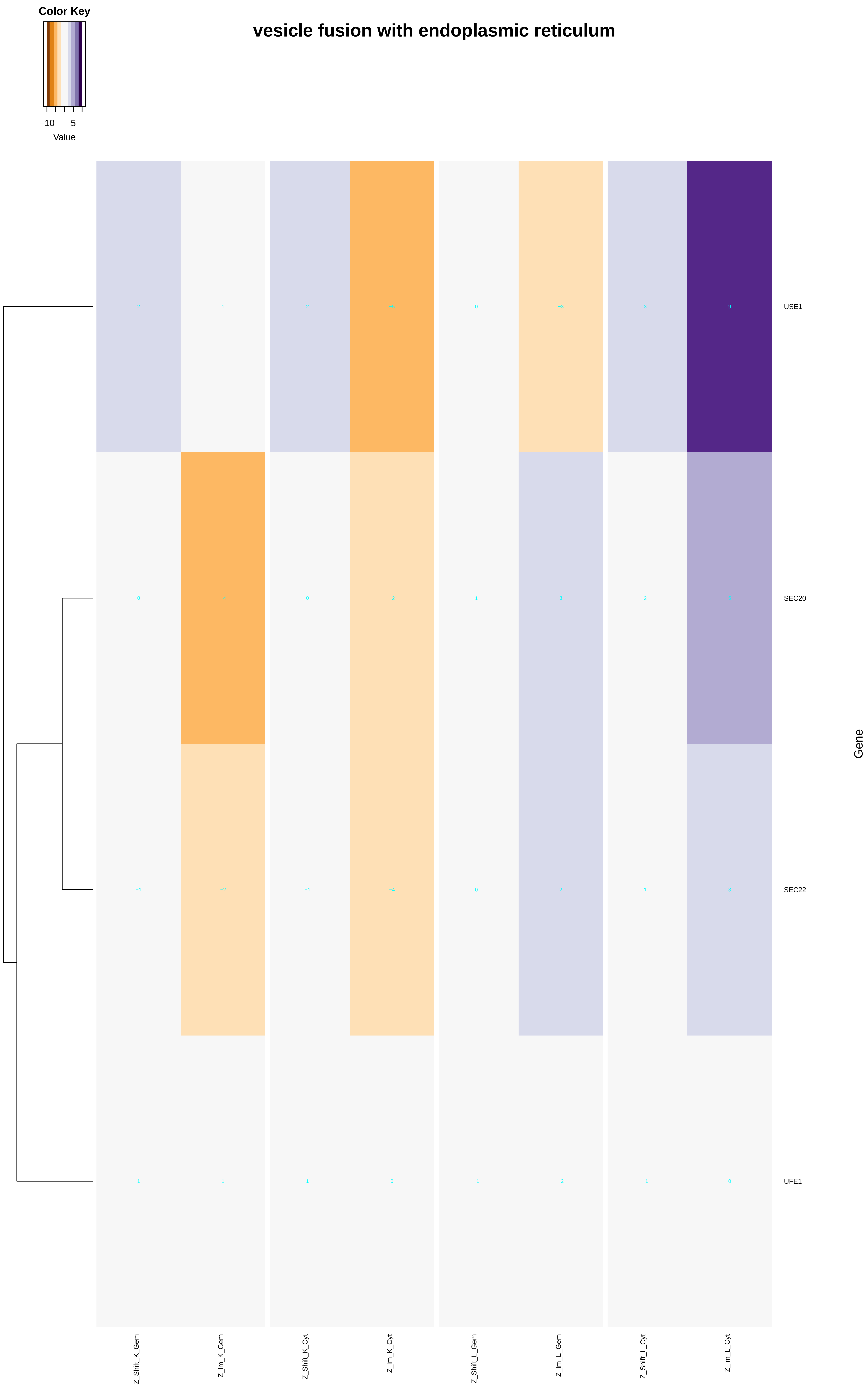
vesicle fusion with endoplasmic reticulum
Color Key
−10
5
Value
USE1
SEC20
SEC22
UFE1
Gene
Z_Shift_K_Gem
Z_Im_K_Gem
Z_Shift_K_Cyt
Z_Im_K_Cyt
Z_Shift_L_Gem
Z_Im_L_Gem
Z_Shift_L_Cyt
Z_Im_L_Cyt

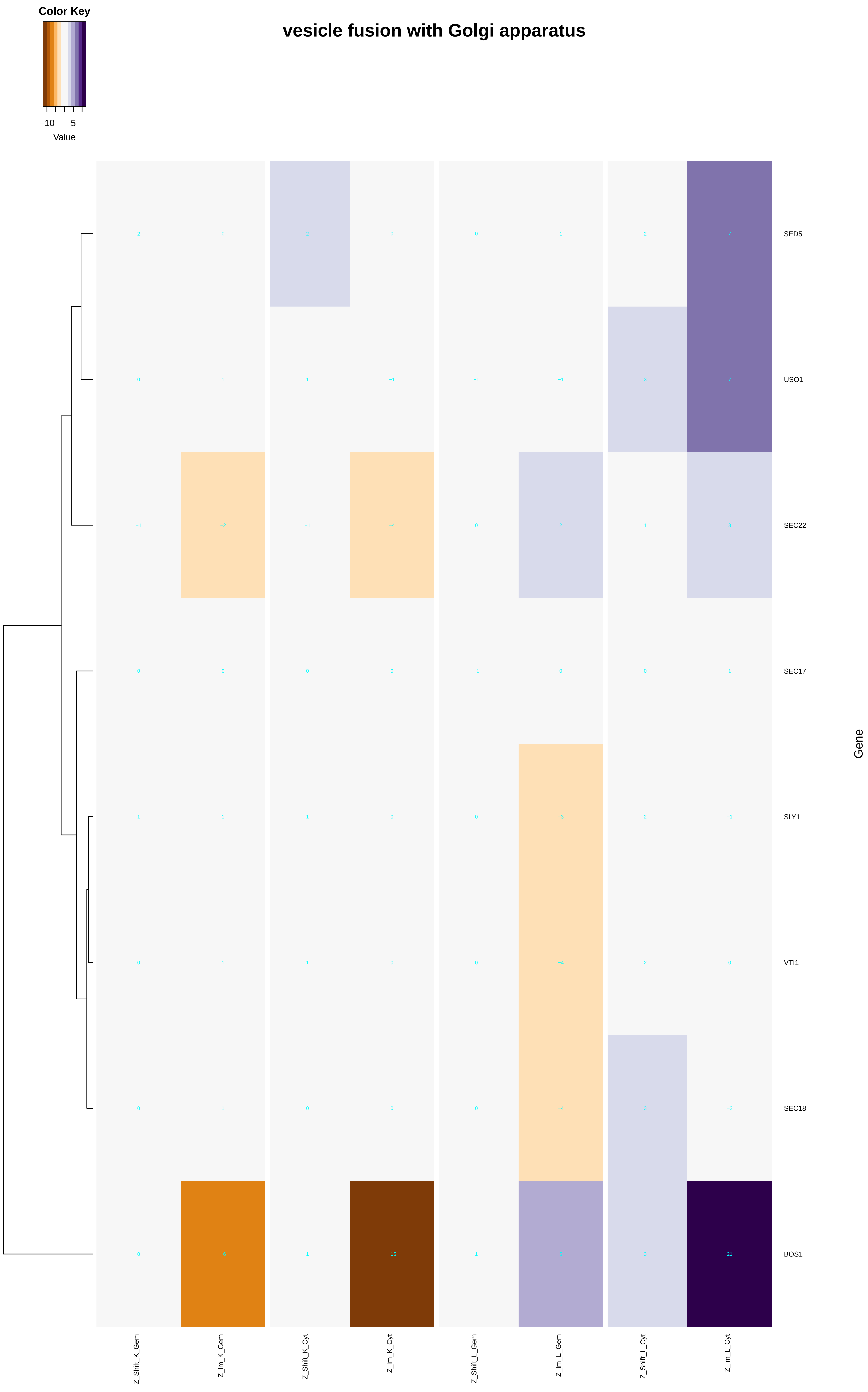

Color Key
−10
5
Value
vesicle fusion with Golgi apparatus
SED5
USO1
SEC22
SEC17
SLY1
VTI1
SEC18
BOS1
Gene
Z_Shift_K_Gem
Z_Im_K_Gem
Z_Shift_K_Cyt
Z_Im_K_Cyt
Z_Shift_L_Gem
Z_Im_L_Gem
Z_Shift_L_Cyt
Z_Im_L_Cyt

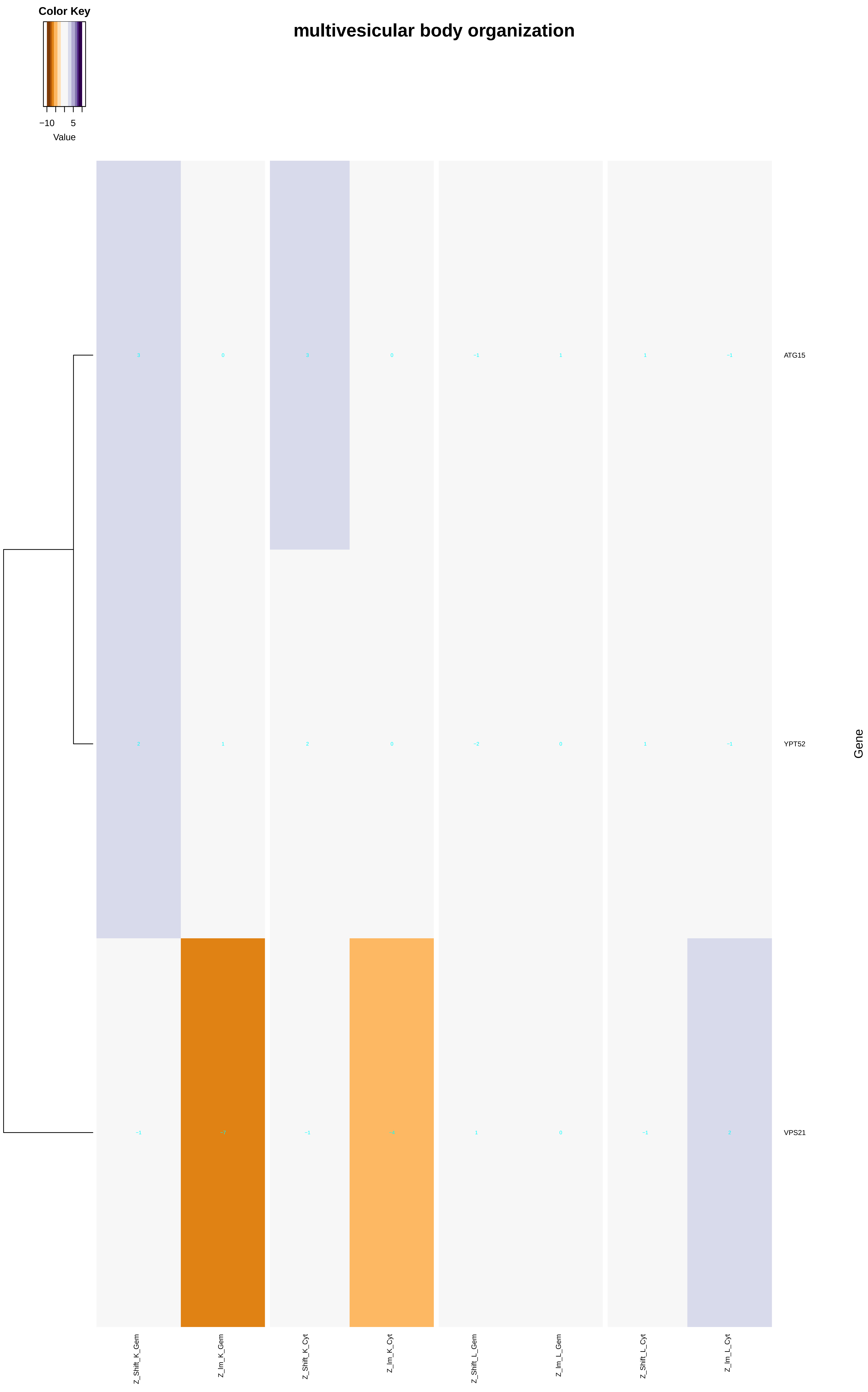

Color Key
−10
5
Value
multivesicular body organization
ATG15
YPT52
VPS21
Gene
Z_Shift_K_Gem
Z_Im_K_Gem
Z_Shift_K_Cyt
Z_Im_K_Cyt
Z_Shift_L_Gem
Z_Im_L_Gem
Z_Shift_L_Cyt
Z_Im_L_Cyt
